# Validation of algorithms for selecting rheumatoid arthritis patients in the Tuscan healthcare administrative databases

**DOI:** 10.1038/s41598-021-98321-0

**Published:** 2021-10-13

**Authors:** Irma Convertino, Massimiliano Cazzato, Sabrina Giometto, Rosa Gini, Giulia Valdiserra, Emiliano Cappello, Sara Ferraro, Silvia Tillati, Claudia Bartolini, Olga Paoletti, Valentina Lorenzoni, Leopoldo Trieste, Matteo Filippi, Giuseppe Turchetti, Michele Cristofano, Corrado Blandizzi, Marta Mosca, Ersilia Lucenteforte, Marco Tuccori

**Affiliations:** 1 Department of Clinical and Experimental Medicine, Unit of Pharmacology and Pharmacovigilance, University of Pisa, Pisa, Italy; 2 Unit of Rheumatology, University Hospital of Pisa, Pisa, Italy; 3 Department of Clinical and Experimental Medicine, Unit of Medical Statistics, University of Pisa, Pisa, Italy; 4 Tuscan Regional Healthcare Agency, Florence, Italy; 5 Institute of Management, Scuola Superiore Sant’Anna, Pisa, Italy; 6 Direzione Medica Di Presidio, University Hospital of Pisa, Pisa, Italy; 7 Unit of Adverse Drug Reactions Monitoring, University Hospital of Pisa, Tuscan Regional Centre of Pharmacovigilance, Via Roma, 55, 56126 Pisa, Italy

**Keywords:** Health care, Medical research, Rheumatology

## Abstract

Validation of algorithms for selecting patients from healthcare administrative databases (HAD) is recommended. This PATHFINDER study section is aimed at testing algorithms to select rheumatoid arthritis (RA) patients from Tuscan HAD (THAD) and assessing RA diagnosis time interval between the medical chart date and that of THAD. A population was extracted from THAD. The information of the medical charts at the Rheumatology Unit of Pisa University Hospital represented the reference. We included first ever users of biologic disease modifying anti-rheumatic drugs (bDMARDs) between 2014 and 2016 (index date) with at least a specialist visit at the Rheumatology Unit of the Pisa University Hospital recorded from 2013 to the index date. Out of these, we tested four index tests (algorithms): (1) RA according to hospital discharge records or emergency department admissions (ICD-9 code, 714*); (2) RA according to exemption code from co-payment (006); (3) RA according to hospital discharge records or emergency department admissions AND RA according to exemption code from co-payment; (4) RA according to hospital discharge records or emergency department admissions OR RA according to exemption code from co-payment. We estimated sensitivity, specificity, positive and negative predicted values (PPV and NPV) with 95% confidence interval (95% CI) and the RA diagnosis median time interval (interquartile range, IQR). Two sensitivity analyses were performed. Among 277 reference patients, 103 had RA. The fourth algorithm identified 96 true RA patients, PPV 0.78 (95% CI 0.70–0.85), sensitivity 0.93 (95% CI 0.86–0.97), specificity 0.84 (95% CI 0.78–0.90), and NPV 0.95 (95% CI 0.91–0.98). The sensitivity analyses confirmed performance. The time measured between the actual RA diagnosis date recorded in medical charts and that assumed in THAD was 2.2 years (IQR 0.5–8.4). In conclusion, this validation showed the fourth algorithm as the best. The time interval elapsed between the actual RA diagnosis date in medical charts and that extrapolated from THAD has to be considered in the design of future studies.

## Introduction

In the last ten years, the use of healthcare administrative databases (HAD) in population-based observational studies largely increased in pharmacoepidemiology research due to several advantages. First of all, since these databases collect information on healthcare services accessed by all patients, including supplying of drugs, they can be very useful for conducting studies with large samples of individuals, also representing the entire population of drug users, with extended follow up periods^1,2^. Second, these databases can be particularly useful for investigating chronic diseases, such as rheumatoid arthritis (RA)^3^. In Italy, since the National and Regional Healthcare Systems cover the majority of costs of medical care of the whole residents, these HAD are particularly suitable for real world data investigations. However, many limitations in study design and interpretation of results must be taken into account. Since data are collected mainly for reimbursement purposes, misclassification and undisclosed confounding should be carefully considered. For instance, one of the main issues of the use of these databases is the lack of indications for supplied drugs. This problem is particularly relevant for drugs with multiple indications. Information on possible indications is sometimes, but not always, recorded only for patients accessing the hospital care in hospital discharge records (International Classification of Diseases, 9th Revision, ICD-9 codes). More often, patients may be recorded with a disease-based exemption code from co-payment, which allows the free access to healthcare facilities, because of the clinical burden of their disease. Unfortunately, there are multiple codes (e.g. there are exemption codes from co-payment that are age-based or income-based) and, for patients with multiple exemption choices from co-payment, there are not priority rules prescribers should select and record. A further complication of the interpretation of results is that, using these proxies, the date of diagnosis captured in HAD and the date of actual diagnosis recorded in medical charts rarely overlap. In this scenario, the best strategy for selecting patients by indication is likely the construction of algorithms that combines the available information. However, since several algorithms can be proposed, a validation study is strongly recommended to support the reliability of their results^2,4^.

The Pathfinder study^5^, uses data collected in the HAD of Tuscany to investigate RA patients exposed for the first time to biologic disease modifying anti-rheumatic drugs (bDMARDs). Given the concerns related to the identification of RA patients mentioned above, the study protocol provides several algorithms to implement the cohort selection strategy. Based on the Tuscan HAD, the present investigation aimed at validating four algorithms in order to select the best one(s) for selecting RA patients, also investigating the time elapsed between the diagnosis date recorded in medical charts and that captured in the HAD.

## Methods

### Study design and data sources

**Figure 1:**
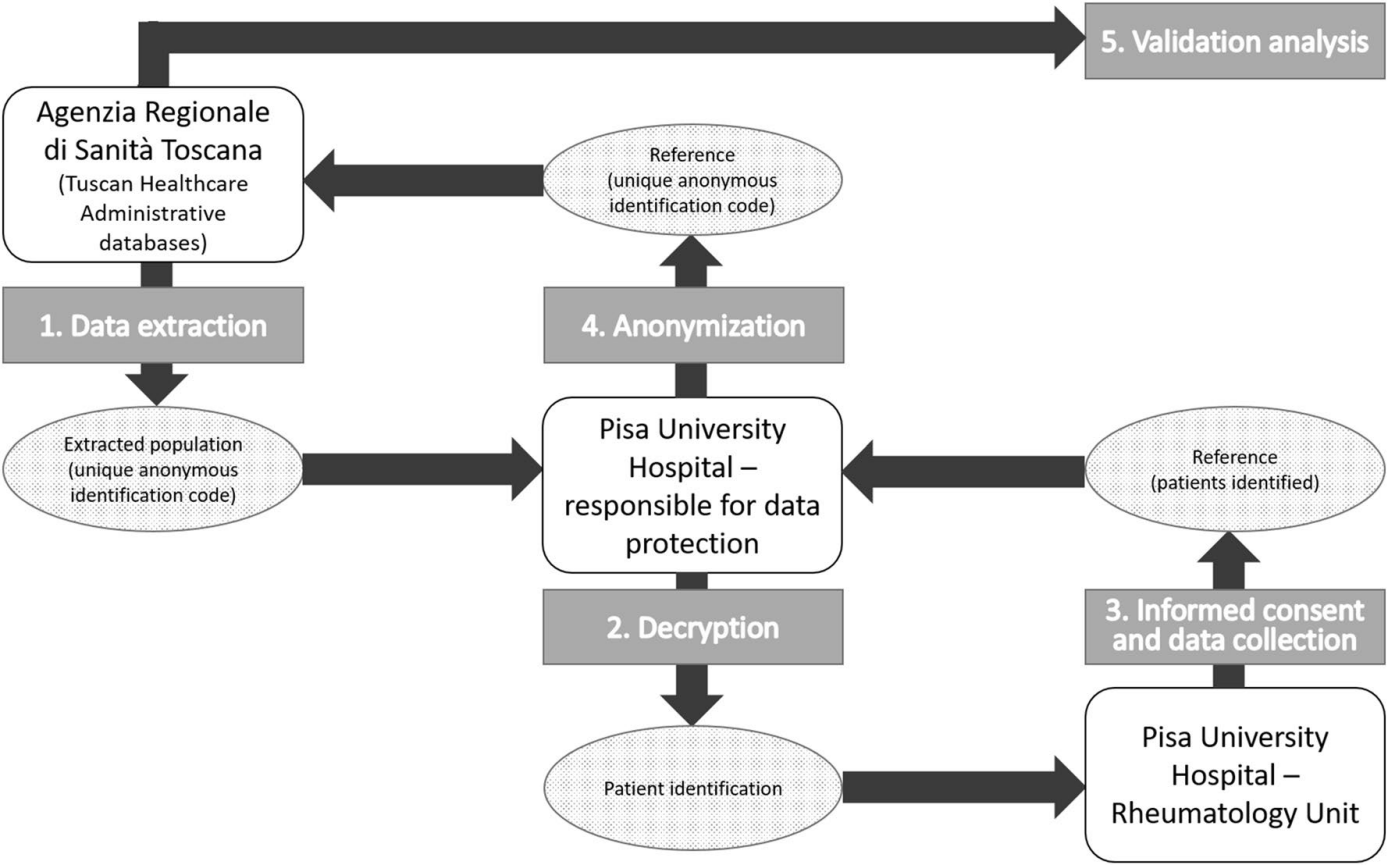
Validation dataflow. 1. The Agenzia Regionale di Sanità Toscana selected from Tuscan Healthcare Administrative databases (THAD) the extracted population through the unique anonymous identification code (UAIC). 2. The list of codes were sent to the responsible for data protection of the Pisa University Hospital for the decryption process of patient codes that consists in associating the corresponding internal ID code. 3. The investigators of the Rheumatology Unit of the Pisa University Hospital acquire the informed consent of the identified patients and collect the clinical data of interest from their medical charts (reference). 4. The reference sample is anonymized again with the UAIC and data collected from medical charts has been linked to data recorded in the THAD. 5. Finally, the validation analysis was performed.

This is a retrospective validation study conducted on a population of patients extracted from the HAD of Tuscany (Italy). The Tuscan population, amounting to about 3.6 millions of inhabitants in 2016, is covered by a national, universal, single-payer, public health system, of which the services dispensed to patients at regional level were recorded in the HAD^6^. The Tuscan HAD comprise data electronically collected since 2004. In particular, for these analyses we extracted data on April 29^th^, 2019 from the following repositories: drug supply to inpatients and outpatients database, exemptions from co-payments database, hospital discharge records, emergency department accesses records, and outpatient services for specialist visits. Hospital discharge records includes information on patient diseases (ICD-9 codes) organized in one primary diagnosis (usually the main cause of hospitalization) and several secondary diagnoses (other patient relevant co-morbidities). Emergency department access registry includes information on the main cause of admissions (ICD-9 codes). Exemption from co-payments codes identify subjects with characteristics (e.g. disease-related, age related, income-related) for which the regional healthcare system provides full coverage of the cost of the services supplied. The pseudo-anonymized information of Tuscan patients contained in these administrative data sources (representing the extracted population) was linked to the data of the corresponding medical chart of the Rheumatology Unit of Pisa University Hospital (representing the reference). The datasheet provided for the analyses included only the patient unique identification number. This number was decrypted, and patient name organized in an alphabetic order by the Hospital Healthcare Office to allow patient identification by the rheumatologist. Patients were then contacted during scheduled visits or by phone to receive their informed consent for participating to the study. From the chart medical records of each of these patients, the following information was collected retrospectively: RA diagnosis, date of RA diagnosis, date of visits. Patients’ data were then anonymized again, and linked to the original datasheet by the unique identification code for the final analysis (Fig. 1).

This study obtained the European Network of Centres for Pharmacoepidemiology and Pharmacovigilance (ENCePP) seal (EUPAS29263)^5^ and the authorization by the Ethical Committee of Pisa University Hospital (Protocol number 18724), as regard the ethical and privacy protection requirements, needed for accessing data of chart medical records. The patient consent was obtained, and de-identified patient records were used in the analyses.

### Study population and patient classification

We have extracted from the regional HAD the population of bDMARDs users receiving their first ever dispensation of one bDMARD (i.e. infliximab, adalimumab, certolizumab pegol, etanercept, golimumab, abatacept, tocilizumab, rituximab) from 2014 to 2016. The date of this first ever dispensation was defined as the index date. Then, we selected those bDMARD users with at least one record of visit at the Rheumatology Unit of the Pisa University Hospital from 2013 to the index date. This population included first ever users of bDMARDs for any indication that have been tracked in the rheumatology setting (extracted population). The corresponding medical charts were identified by using the patient unique identification number to create the reference population. The information of diagnosis collected in medical charts (reference) was used to identify the actual RA and non-RA patients. In particular, the actual diagnosis of RA used as reference was that recorded in the medical chart by the rheumatologist, based on patient clinical assessment. The date of the visit in which the diagnosis of RA was recorded in the medical chart was used as the date of the actual diagnosis. Since the diagnosis of RA can be retrieved from the medical charts only and not from HAD, we used the earliest RA diagnosis recorded in the hospital discharges (regardless of primary or secondary) and emergency department admissions or the co-payment exemption code related to RA as a proxy of the RA diagnosis for HAD (assumed diagnosis). The date of hospital discharge or emergency department admission or assignment of a co-payment exemption code in which the assumed diagnosis of RA was recorded, whichever came first, was used as the date of the assumed diagnosis.

We tested the performance of four algorithms in the identification of true positive patients (i.e. patients with assumed RA diagnosis according to index test that were actual RA patients according to the reference) and true negative patients (i.e. patients with assumed non-RA diagnosis according to index test that were actually non-RA patients according to reference). Out of patients with the first supply of bDMARD from 2014 to 2016 and at least one visit at the Rheumatology Unit of Pisa University Hospital from 2013 to the index date, we tested the performance of the following four index tests (algorithms): (1) RA according to hospital discharge records or emergency department admissions (ICD-9 code, 714*); (2) RA according to exemption code from co-payment (006); (3) RA according to hospital discharge records or emergency department admissions (ICD-9 code, 714*) AND RA according to exemption code from co-payment (006); 4) RA according to hospital discharge records or emergency department admissions (ICD-9 code, 714*) OR RA according to exemption code from co-payment (006) (Supplementary Material, SM Fig. 1).

### Data analysis

We estimated the sensitivity (proportion of true positive RA patients over the number of actual RA patients according to reference); specificity (proportion of true negative RA patients over the number of actual non-RA patients according to reference); positive predictive value, PPV, (proportion of true positive RA patients over all patients classified as RA according to the algorithm) and negative predictive value, NPV, (proportion of true negative RA patients over all patients classified as non-RA according to the algorithm), and the corresponding 95% confidence intervals (CIs) for each algorithm. Patients with missing information of diagnosis in the reference were classified as non-RA patients. These were included in the main analysis. Then, to evaluate whether they could have affected the performance of algorithms found, we performed a first sensitivity analysis where we excluded patients with missing diagnosis from the reference. The second sensitivity analysis was carried out by testing the four algorithms according to the patients’ age, in order to test the effect of the competing age-based exemption code from co-payment. This stratification involved two groups: < 65 years old and ≥ 65 years old. In the three analyses, we ranked the algorithms by using the Youden index^7^.

Finally, for the true positive patients and for each algorithm, the median time (interquartile range, IQR) elapsed from the date of actual RA diagnosis in the medical charts and the earliest date of assumed RA diagnosis as captured in the HAD was estimated.

All analyses were performed on de-identified data using R, version 3.6.3.

### Ethics approval

This retrospective chart review study involving human participants was in accordance with the ethical standards of the institutional and national research committee and with the 1964 Helsinki Declaration and its later amendments or comparable ethical standards. The Ethical Committee of the Pisa University Hospital approved this study (Protocol number 18724).

### Consent to participate

Informed consent was obtained from all individual participants included in the study.

### Consent for publication

All patients were required to give their consent to the publication of study results.

## Results

Out of 288 patients in the extracted population, 277 gave their consent to participate to our study. In the reference, 103 patients were RA patients and 21 had missing data of interest. In HAD, for 114 patients a RA diagnosis was retrieved from hospital discharges, emergency department admissions, and exemption from co-payments, and age information was also available. Out of these, 72 patients (63.2%) had diagnosis between 41 and 65 years old and the mean age at diagnosis was 53.3 (standard deviation, SD 13.9). Furthermore, the mean time occurred from the assumed RA diagnosis to the first bDMARD was 5.8 (SD 5.4) years. We observed also that few patients (n = 14) have had a dispensation of the first bDMARD before the assumed RA diagnosis amounting to 1.8 years as mean (SD 1.8).

**Figure 2:**
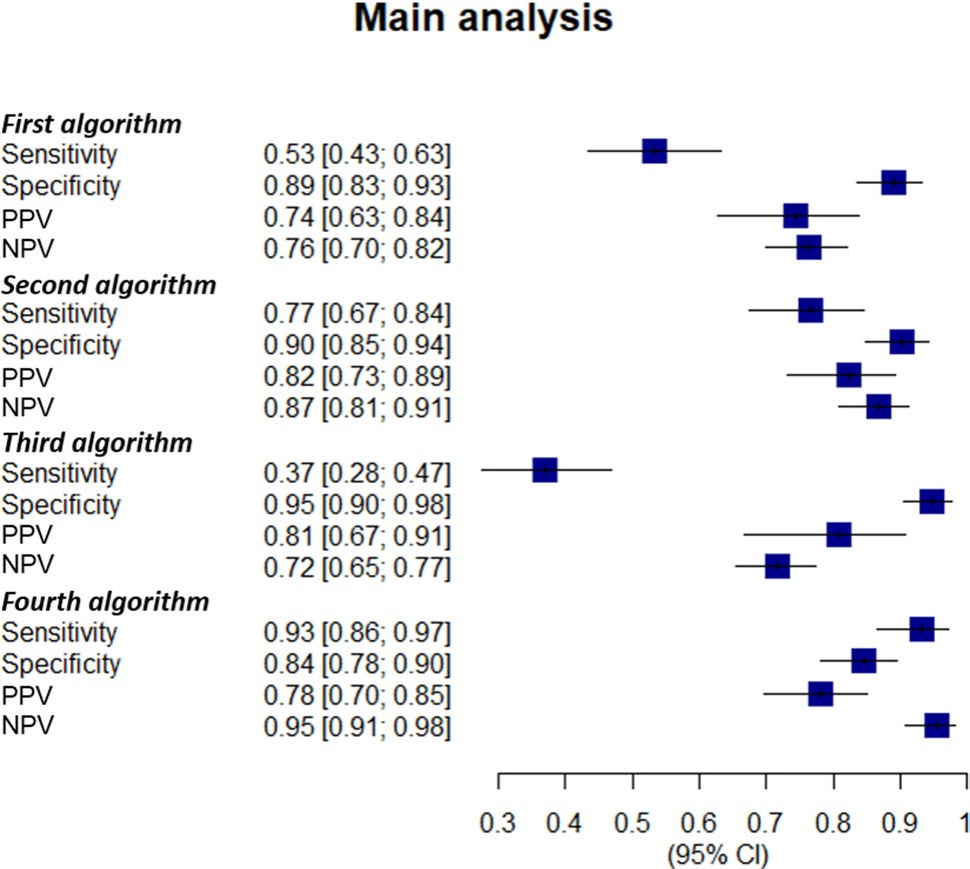
Validation of the algorithms used for selecting rheumatoid arthritis patients: the main analysis. The four algorithms were evaluated for: sensitivity (proportion of patients correctly classified as rheumatoid arthritis (RA) patients by the algorithm within the RA ones); specificity (proportion of patients correctly classified as without RA by the algorithm within patients without RA); positive predictive value (proportion of patients correctly classified as RA by the algorithm within all patients classified as RA by the algorithm) and negative predictive value (proportion of patients correctly classified as without RA by the algorithm within all patients classified as non-RA by the algorithm). Out of patients with the first bDMARD supply from 2014 to 2016 and at least one visit at the Rheumatology Unit of Pisa University Hospital from 2013 to the index date, the four algorithms involved the following items: (1) RA according to hospital discharge records or emergency department admissions (ICD-9 code, 714*); (2) RA according to exemption code from co-payment (006); (3) RA according to hospital discharge records or emergency department admissions (ICD-9 code, 714*) AND RA according to exemption code from co-payment (006); (4) RA according to hospital discharge records or emergency department admissions (ICD-9 code, 714*) OR RA according to exemption code from co-payment (006). *bDMARD* biologic disease modifying anti-rheumatic drugs, *95% CI* 95% confidence interval, *ICD-9* international classification of diseases 9th revision, *NPV* negative predictive value, *PPV* positive predictive value, *RA* rheumatoid arthritis.

Figure 2 displays the results of the main analysis. Overall, the four algorithms showed good PPV, sensitivity, specificity and NPV (> 0.70) with the exception of the two algorithms including the RA diagnosis captured in hospital discharge records and emergency department accesses. Indeed, sensitivity values of 0.53 (95% CI 0.43–0.63) and 0.37 (95% CI 0.28–0.47) were found for the first and third algorithm, respectively. The best algorithm observed, after ranking, was the fourth one, made up of RA diagnosis according to hospital discharge records or emergency department admissions OR RA according to disease exemption code from co-payment (SM Table 1). This was able to select 96 true positive patients (Table 1). The following values were found: PPV 0.78 (95% CI 0.70–0.85), sensitivity 0.93 (95% CI 0.86–0.97), specificity 0.84 (95% CI 0.78–0.90), and NPV 0.95 (95% CI 0.91–0.98). The SM Tables 2–5 showed the distribution of the true positive and true negative according to the four algorithms.

**Table 1:** Distribution of RA patients selected through the four algorithms: the main analysis.

Algorithms*	Actual RA patients^°^, *n (%)*	Assumed RA patients^§^, *n (%)*	True positive patients^#^, *n (%)*
First	103 (37.2)	74 (71.8)	55 (53.4)
Second	103 (37.2)	96 (93.2)	79 (76.7)
Third	103 (37.2)	47 (45.6)	38 (36.9)
Fourth	103 (37.2)	123 (119.4)	96 (93.2)

*Out of patients with the first supply of bDMARD from 2014 to 2016 and at least one record of visit at the Rheumatology Unit of Pisa University Hospital from 2013 to the index date, we tested the performance of four index tests (algorithms):

First) RA according to hospital discharge records or emergency department admissions (ICD-9 code, 714*);

Second) RA according to exemption code from co-payment (006);

Third) RA according to hospital discharge records or emergency department admissions (ICD-9 code, 714*) AND RA according to exemption code from co-payment (006);

Fourth) RA according to hospital discharge records or emergency department admissions (ICD-9 code, 714*) OR RA according to exemption code from co-payment (006).

°The actual diagnosis of RA was that recorded in the medical chart (reference).

^§^the assumed diagnosis was the RA diagnosis recorded in the hospital discharges (regardless of primary or secondary) and emergency department admissions or the co-payment exemption code related to RA in HAD.

^#^True positive patients: These were assumed RA patients in the HAD with actual RA diagnosis in the reference.

*bDMARD* biologic disease modifying antirheumatic drug, *HAD* Healthcare Administrative Database, *ICD-9* international classification of diseases 9th revision, *n* number; *RA* rheumatoid arthritis.

**Figure 3:**
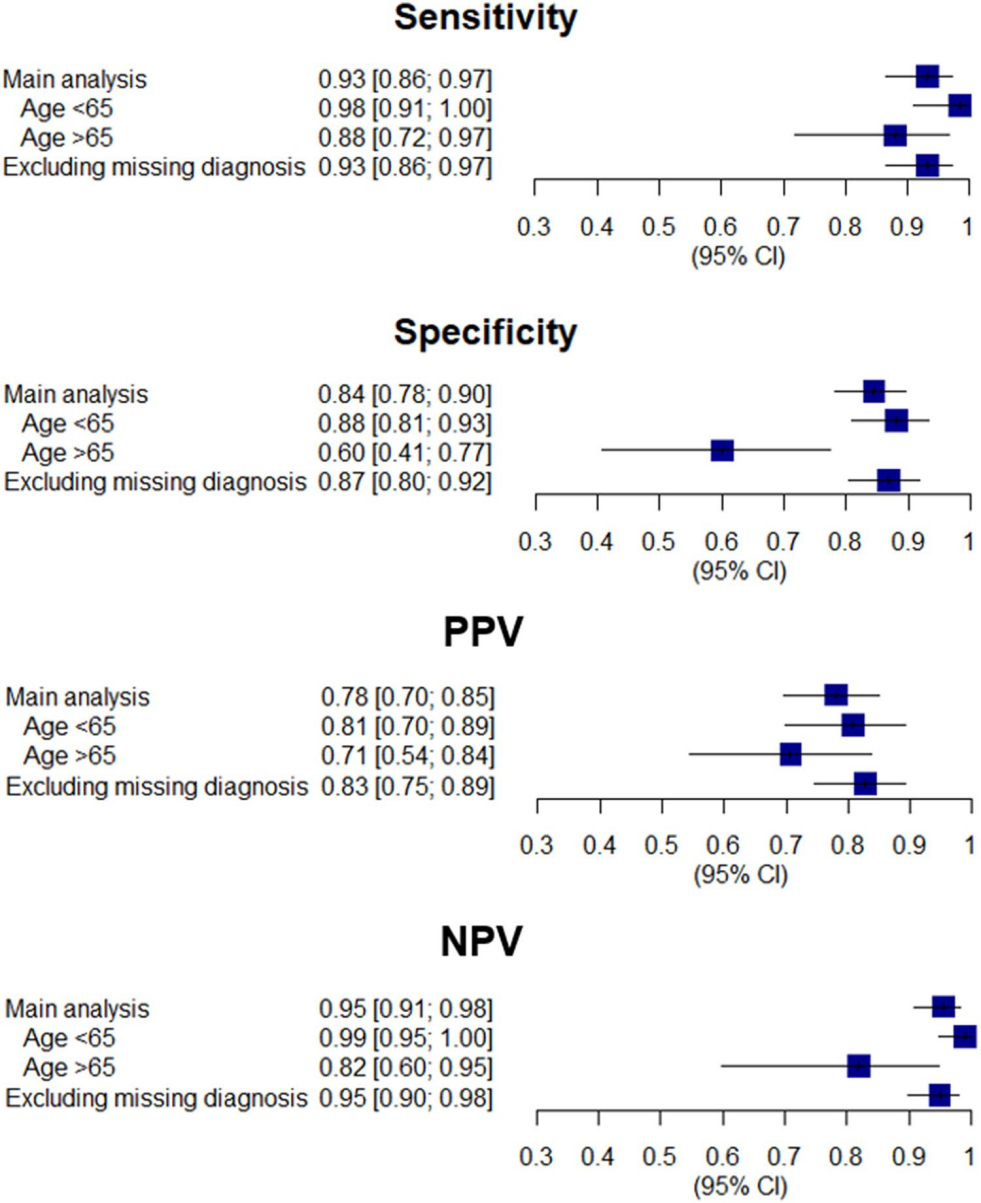
Estimations of the fourth algorithm in the three analyses. The fourth algorithm was made up of RA according to hospital discharge records or emergency department admissions (ICD-9 code, 714*) OR RA according to exemption code from co-payment (006). Sensitivity (percentage of patients rightly classified as having rheumatoid arthritis (RA) by the algorithm within the RA patients); specificity (percentage of patients rightly classified as non-having RA by the algorithm within non-RA patients); positive predictive value (percentage of patients rightly classified as RA by the algorithm within all patients classified as RA by the algorithm) and negative predictive value (proportion of patients rightly classified as non-RA by the algorithm within all patients classified as non-RA by the algorithm) were calculated in the three analyses. The main analysis included all patients in the reference sample even those with missing diagnosis, classified as non-RA patients. The first sensitivity analysis excluded patients with missing diagnosis in the reference. The second sensitivity analysis stratified patients according age into two groups: patients under 65 years old and patients over 65 years. *95% CI* 95% confidence interval, *NPV* negative predictive value, *PPV* positive predictive value, *RA* rheumatoid arthritis.

The first sensitivity analysis confirmed the robustness of findings of the main analysis. The second sensitivity analysis showed that in the group of patients under 65 years old, all estimations increased for the four algorithms. On the contrary, these values decreased for patients aged ≥ 65 years (SM Figs. 2–4). In particular, the sensitivity rose to 0.98 for the fourth algorithm including the disease exemption from co-payment or the RA diagnosis in combination with the visit and the bDMARD when patients younger than 65 years old were considered, and values over 0.80 were observed for the remaining estimations in this group (Fig. 3).

Out of the 96 true positive patients identified by the fourth algorithm, 68 reported an available date of RA diagnosis in the medical charts. The 89.7% (n = 61) of these latter had a date of assumed RA diagnosis subsequent to that of the actual diagnosis. The median time elapsed between these two dates is 2.2 years (IQR 0.5–8.4). In addition, 7.3% (n = 7) of these patients had a diagnosis of assumed RA reported earlier than the actual RA diagnosis with a median elapsed period between these two of − 2.0 years (IQR − 7.4 to − 1.3) (Table 2).

**Table 2:** Median time elapsed between the date of assumed rheumatoid arthritis diagnosis recorded in the administrative databases and the actual one in the medical charts.

Algorithms*	Patients^a^, *n (%)*	Patients^b^, *n (%)*	Years, *median (IQR)*
First	5 (12.5)		− 2.0 (−5.4 to − 1.9)
Second	4 (7.5)		− 2.8 (−6.1 to − 0.7)
Third	2 (8.0)		− 3.0 (−4.2 to − 1.9)
Fourth	7 (10.3)		− 2.0 (− 7.4 to − 1.3)
First		35 (87.5)	7.6 (3.3 to 16.2)
Second		49 (92.5)	1.8 (0.5 to 4.0)
Third		23 (92.0)	4.9 (2.8 to 10.6)
Fourth		61 (89.7)	2.2 (0.5 to 8.4)

*Out of patients with the first supply of bDMARD from 2014 to 2016 and at least one record of visit at the Rheumatology Unit of Pisa University Hospital from 2013 to the index date, we tested the performance of four index tests (algorithms):

First) RA according to hospital discharge records or emergency department admissions (ICD-9 code, 714*);

Second) RA according to exemption code from co-payment (006);

Third) RA according to hospital discharge records or emergency department admissions (ICD-9 code, 714*) AND RA according to exemption code from co-payment (006);

Fourth) RA according to hospital discharge records or emergency department admissions (ICD-9 code, 714*) OR RA according to exemption code from co-payment (006).

^a^Patients with RA diagnosis recorded firstly in the administrative database.

^b^Patients with RA diagnosis recorded firstly in the medical charts.

*IQR* interquartile range, *n* number, *RA* rheumatoid arthritis.

## Discussion

Although HAD contain large amounts of data that can be useful for answering to specific research questions with important clinical implications, the quality of the source and/or the choice of the wrong selection criteria could compromise the reliability of results. The present study findings showed that, using the best algorithm, the HAD of Tuscany could be a good data source for performing population-based studies on RA patients. All the four algorithms tested were able with different performance to select true positive patients by identifying RA patients in the HAD, who matched the RA ones as defined in the medical charts. Based on this, we found that the fourth algorithm provided the most reliable results matching the majority of true RA patients (93.2%). Of note, the use of the disease exemption code from co-payment as proxy of RA diagnosis appears to be the most reliable variable when compared with the ICD-9 code reported in hospital discharge records or emergency department accesses. This could be explained, on one side, by the condition that RA patients could have a higher probability to be associated with the disease exception code from co-payment than hospitalizations or emergency department admissions and, on the other, by the nature of the data source used. Indeed, in Italy the HAD, including the Tuscan ones, were employed first for reimbursement purposes. Overall, when secondary data are used in research, the selection of the most suitable codes to detect variables of exposure and outcome is essential for performing high quality studies^8^. Albeit data collected in the administrative repositories appears accurate^9^, the possibility of misclassifications has to be taken into account during both the study design and the interpretation of results. Other issues intrinsic with the nature of the databases must be considered. For instance, reimbursement reasons can lead to over- or under-coding of certain conditions in the administrative databases favouring the reporting of serious and severe events more frequently than that not serious and with less reimbursement rate^10^. In addition, the age strictly affects the ability of algorithms to select RA patients through the codes of diagnosis or disease exemption. In particular, findings of the second sensitivity analysis display that younger age is associated with the highest sensibility when using the disease exemption from co-payment than the older one. Therefore, our best algorithm reached very good values for all estimations, ranging between 70% and 100%, in patients aged less than 65 years. To the best of our knowledge, this is the first study attempting an estimation of the time elapsing between the actual diagnosis of RA and that assumed in HAD. When we investigated the median time needed by the Tuscan HAD to capture the first information about the RA diagnosis, we found that, among RA patients selected by the best algorithm identified, this median period was of 2.2 years. In our opinion, this information will be very helpful for the design of future studies and the interpretation of their results. Out of these, few true RA patients have a RA diagnosis recorded earlier in the HAD than in the medical charts. This could be the case of patients that have undertaken biologic drugs for other immune mediated inflammatory conditions (IMIDs), whose diagnosis occurred earlier than RA. This possibility is not uncommon since RA patients frequently had history of Crohn's disease, ulcerative colitis or psoriasis^11,12^. Moreover, this could also explain the dispensation of the first bDMARD before diagnosis of RA, observed in some patients.

Our best performing algorithm can not be automatically used to identify RA patients in other Italian regional databases, given the existing heterogeneity of the local healthcare services. In line with this, comparing other studies and validating algorithms could help to better identify RA patients. Such validation studies, conducted in Countries other than Italy, found also good performances for the algorithms tested^13–17^, particularly when the detection of RA diagnosis is associated with the prescription of at least one DMARD^13–15,17^. Noteworthy, by comparing all estimations of these studies with ours, the best algorithm found in the present study showed the highest sensitivity (i.e., over 90%), resulting in a good performance in capturing the true RA patients in our database. To the best of our knowledge, only another validation study has been carried out on the identification of RA patients in Italy^18^. Carrara et al.^18^, tested an algorithm for selecting RA patients in Lombardy, by including among variables the RA ICD-9 code captured in local HAD and the supply of several DMARDs, with results around 80% for all values. Thus, by comparing available evidence, our best algorithm should provide very reliable results in studies requiring the identification of RA patients performed in Tuscan HAD. However, we should consider that not all RA patients could have had a prescription of a biologic drug and a specialist visit in their clinical history. Therefore, from the RA population in Tuscany to the more general case of Italy^16^, the accuracy of our algorithm is expected to vary in relationship with the degree of representativeness of our sample. Particularly, the performance of such algorithms is influenced by the prevalence of RA in the reference sample^3^.

This study has limitations. First, by selecting patients for the reference by their access to RA ward and with a supply of a bDMARD, our algorithm likely does not allow to capture all RA patients but only those with moderate to severe RA. Second, for some patients included in the reference, the quality of information reported in the medical charts did not allow to identify a specific diagnosis. This could lead to an overestimation or an underestimation of the performance of the algorithms. However, by excluding these patients from the reference, the sensitivity analysis seems to confirm the robustness of our results.

Some points of strength have also to be considered. First, in validation study of AHD the use of a reference from a specialty clinic it is well known to elevate PPV value and to limit the generalizability of the results to the general population. This is because the prevalence of the disease is much higher in patients receiving continuous clinical based care^19^. However, a study by Widdifield and coworkers^16^ in RA patients demonstrated that this difference could be very small when the algorithm includes an elevated number of diagnosis codes and musculoskeletal specialist codes for RA. With these premises, it is likely that the PPV of our best performing algorithm is overestimated, although its performance when applied in the general population should be likely slightly reduced, since we have used an elevated number of diagnosis codes for RA. A further improvement could be likely achieved if rheumatology visits could be added to the criteria regardless of the hospital in which they had taken place. Second, the second sensitivity analysis displays how the performance of algorithms for identifying true positive patients is strictly influenced by age when the disease exemption code from co-payment was included among items. Third, the time elapsed between the assumed RA diagnosis in HAD and the actual RA diagnosis of medical charts is a very useful result that has to be taken into account when designating future studies and interpreting related findings.

## Conclusions

In this study, we have identified and validated an algorithm for the identification of RA patients in the Tuscan HAD with very good estimates of sensitivity, specificity, PPV and NPV. Among the variables considered in the tested algorithms, the inclusion of the disease exemption code from co-payment showed to be the most reliable for the identification of RA patients. Our study showed that HAD have a median latency time in the identification of RA diagnosis of about 2 years. This information should be taken into account for future investigations. Our algorithm will be a valuable tool to support the use of the Tuscan HAD for the conduction of further safety and effectiveness study on RA patients and their treatments in the future.

## Supplementary Information

Supplementary Information.
