# Supplementary material for: Validation of algorithms for selecting rheumatoid arthritis patients in the Tuscan healthcare administrative databases
Source: Sci Rep. 2021 Oct 13;11:20314. doi: 10.1038/s41598-021-98321-0 (PMC8514437; doi:10.1038/s41598-021-98321-0)
Supplement: Supplementary file 1 — Supplementary Information. [file 41598_2021_98321_MOESM1_ESM.pdf]

Validation of algorithms for selecting rheumatoid arthritis patients in the Tuscan healthcare administrative databases

Irma Convertino[1§], Massimiliano Cazzato[2§], Sabrina Giometto[3], Rosa Gini[4], Giulia Valdiserra[1], Emiliano Cappello[1], Sara Ferraro[1], Silvia Tillati[3], Claudia Bartolini[4], Olga Paoletti[4], Valentina Lorenzoni[5], Leopoldo Trieste[5], Matteo Filippi[6], Giuseppe Turchetti[5], Michele Cristofano[6], Corrado Blandizzi[1,7], Marta Mosca[2], Ersilia Lucenteforte[3], Marco Tuccori[1,7*]

1 Unit of Pharmacology and Pharmacovigilance, Department of Clinical and Experimental Medicine, University of Pisa, Pisa, Italy
2 Unit of Rheumatology, University Hospital of Pisa, Pisa, Italy
3 Unit of Medical Statistics, Department of Clinical and Experimental Medicine, University of Pisa, Pisa, Italy
4 Tuscan Regional Healthcare Agency, Florence, Italy
5 Institute of Management, Scuola Superiore Sant'Anna, Pisa, Italy
6 Direzione Medica di Presidio, University Hospital of Pisa
7 Unit of Adverse Drug Reactions Monitoring, University Hospital of Pisa, Pisa, Italy

***Corresponding author**

Marco Tuccori, Pharm D PhD
Unit of Adverse Drug Reactions Monitoring
University Hospital of Pisa
Tuscan Regional Centre of Pharmacovigilance
Via Roma, 55, 56126 Pisa, Italy
Tel: +39-050-2218761
Fax: +39-050-992071
E-mail: m.tuccori@ao-pisa.toscana.it; marco.tuccori@gmail.com

[§] co-first author

Supplementary Material (SM) Table 1 Youden index values

Analyses	Algorithms*			
	First	Second	Third	Fourth
Main	0.42	0.67	0.32	0.78
First sensitivity§	0.44	0.70	0.34	0.80
Second sensitivity°				
<65 years	0.41	0.86	0.40	0.86
>65 years	0.21	0.47	0.20	0.48

Out of patients with the first supply of bDMARD from 2014 to 2016 and at least one record of visit at the Rheumatology Unit of Pisa University Hospital from 2013 to the index date, we tested the performance of four index tests (algorithms): First) RA according to hospital discharge records or emergency department admissions (ICD-9 code, 714*); Second) RA according to exemption code from co-payment (006); Third) RA according to hospital discharge records or emergency department admissions (ICD-9 code, 714*) AND RA according to exemption code from co-payment (006); Fourth) RA according to hospital discharge records or emergency department admissions (ICD-9 code, 714*) OR RA according to exemption code from co-payment (006)

§The first sensitivity analysis excluded patients with missing diagnosis in the reference.

°The second sensitivity analysis stratified patients according to age into two groups: patients under 65 years old and patients over 65 years

SM Table 2 Distribution of rheumatoid arthritis diagnosis between the extracted population and the reference according the first algorithm

First algorithm	Reference		Overall
	+ (actual RA patients)	- (actual non-RA patients)	
Extracted population			
+ (assumed RA patients)	TP: 55	FP: 19	TP+FP: 74
- (assumed non-RA patients)	FN: 48	TN: 155	FN+TN: 203
Overall	TP+FN: 103	FP+TN: 174	TOT.: 277

TP: True positive patients; FP: false positive patients; FN: false negative patients; TN: true negative patients; TOT: total

SM Table 3 Distribution of rheumatoid arthritis diagnosis between the extracted population and the reference according the second algorithm

Second algorithm	Reference		Overall
	+ (actual RA patients)	- (actual non-RA patients)	
Extracted population			
+ (assumed RA patients)	TP: 79	FP: 17	TP+FP: 96
- (assumed non-RA patients)	FN: 24	TN: 157	FN+TN: 181
Overall	TP+FN: 103	FP+TN: 174	TOT.: 277

TP: True positive patients; FP: false positive patients; FN: false negative patients; TN: true negative patients; TOT: total

SM Table 4 Distribution of rheumatoid arthritis diagnosis between the extracted population and the reference according the third algorithm

Third algorithm	Reference		Overall
	+ (actual RA patients)	- (actual non-RA patients)	
Extracted population			
+ (assumed RA patients)	TP: 38	FP: 9	TP+FP: 47
- (assumed non-RA patients)	FN: 65	TN: 165	FN+TN: 230
Overall	TP+FN: 103	FP+TN: 174	TOT.: 277

TP: True positive patients; FP: false positive patients; FN: false negative patients; TN: true negative patients; TOT: total

SM Table 5 Distribution of rheumatoid arthritis diagnosis between the extracted population and the reference according the fourth algorithm

Fourth algorithm	Reference		Overall
	+ (actual RA patients)	- (actual non-RA patients)	
Extracted population			
+ (assumed RA patients)	TP: 96	FP: 27	TP+FP: 123
- (assumed non-RA patients)	FN: 7	TN: 147	FN+TN: 154
Overall	TP+FN: 103	FP+TN: 174	TOT.: 277

TP: True positive patients; FP: false positive patients; FN: false negative patients; TN: true negative patients; TOT: total

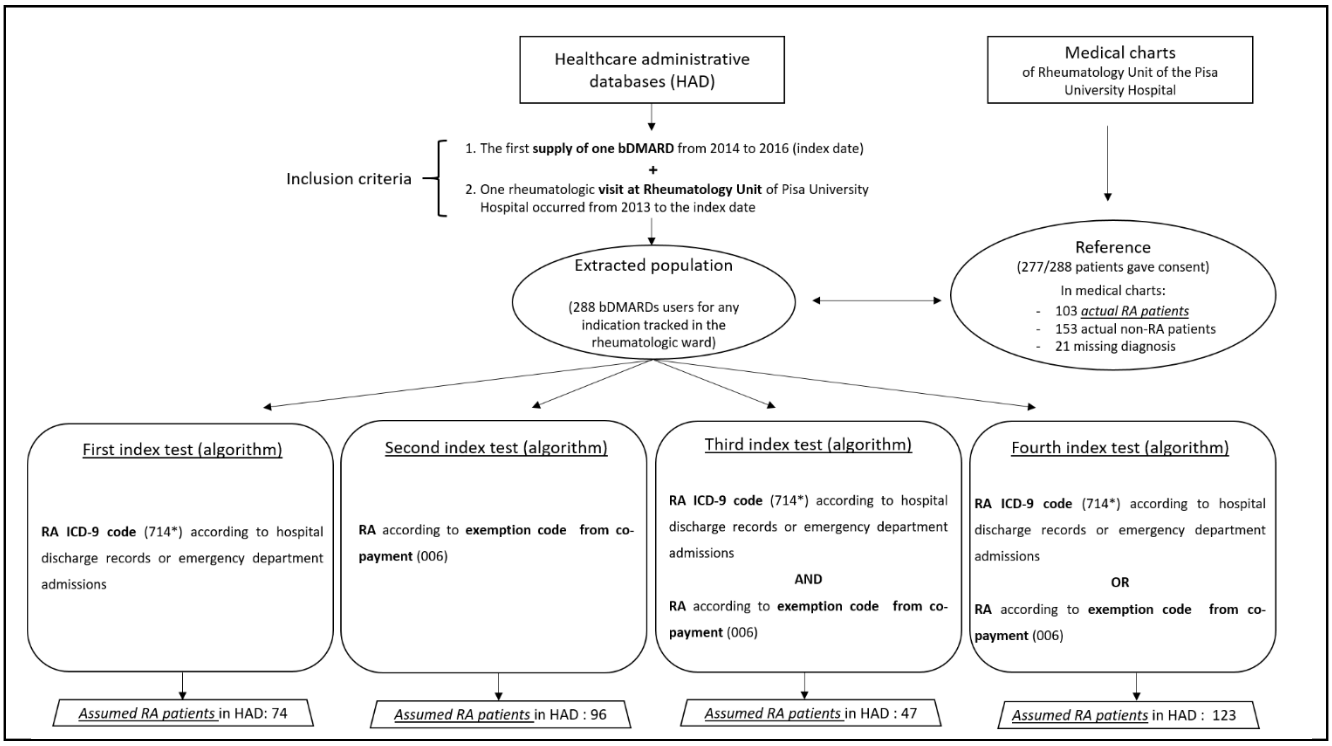

SM Figure 1 Study flow chart

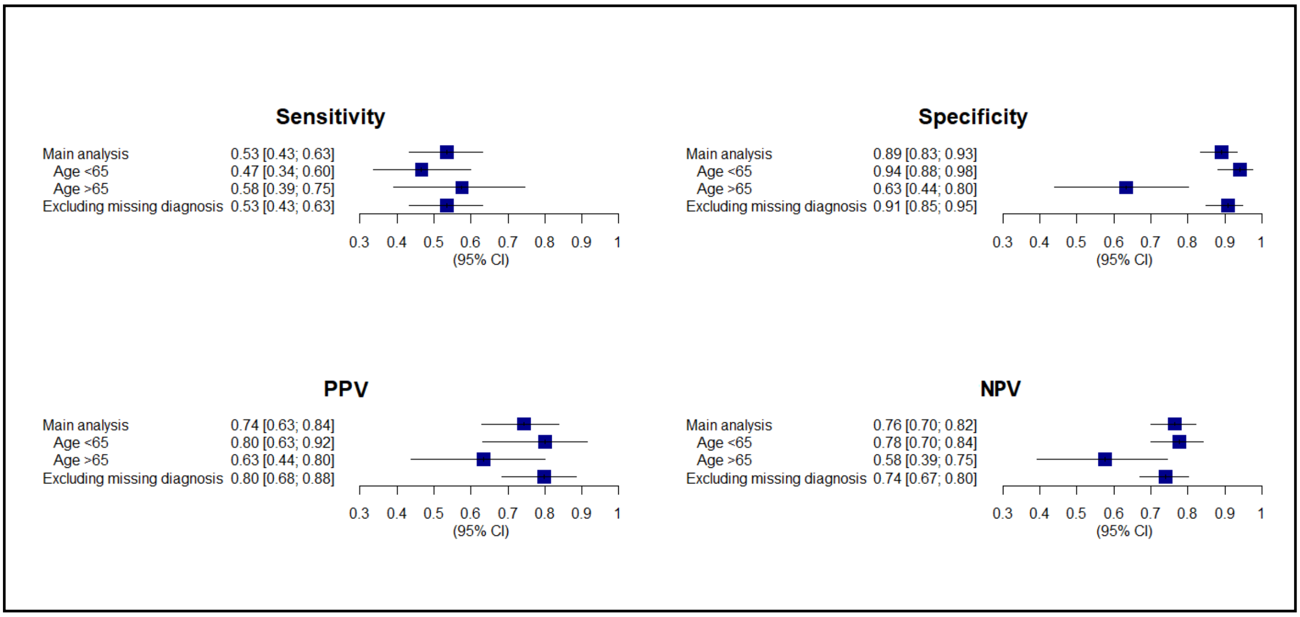

SM Figure 2 Estimations of the first algorithm in the three analyses

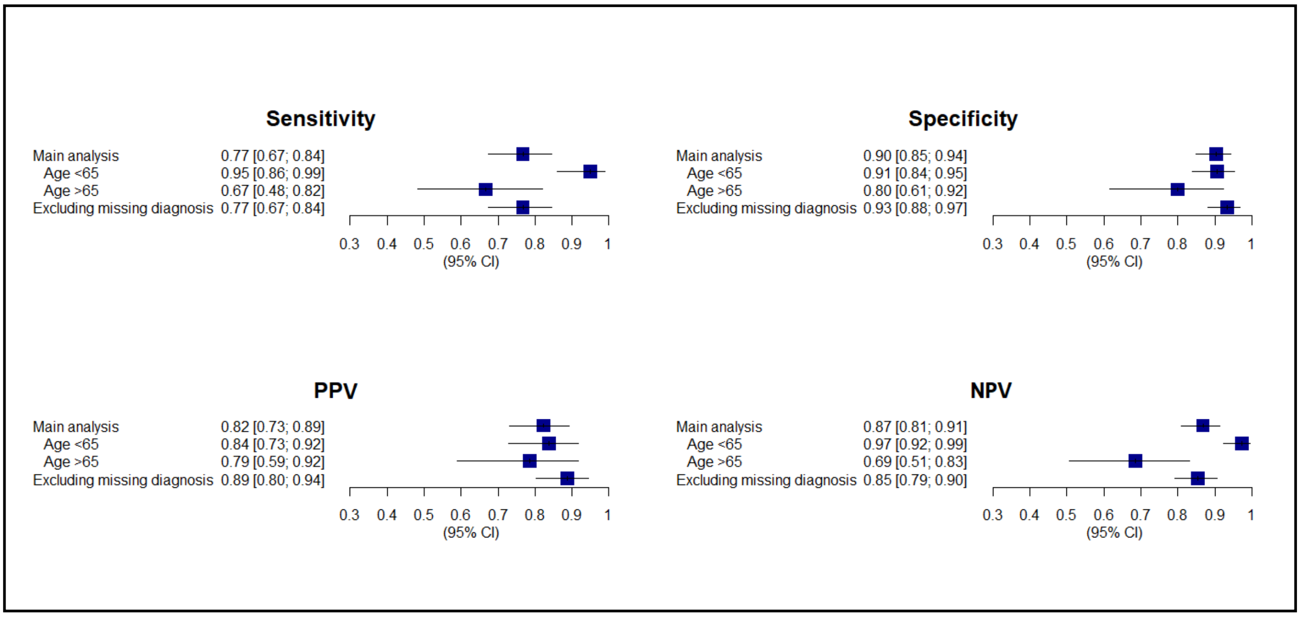

SM Figure 3 Estimations of the second algorithm in the three analyses

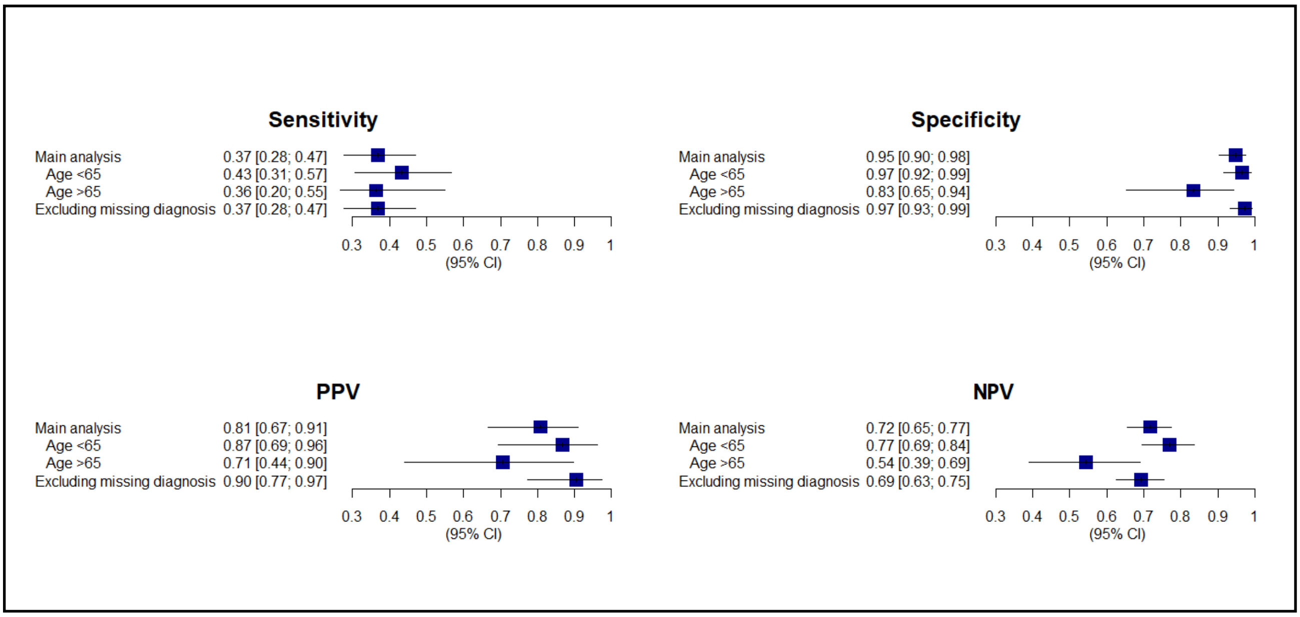

SM Figure 4 Estimations of the third algorithm in the three analyses

Figure captions

Supplementary Material (SM) Figure 1 Study flow chart

Since we have no information about the therapeutic indication of drugs in healthcare administrative database (HAD), we selected patients with the first dispensation of a biologic disease modifying anti-rheumatic drug (bDMARD) and a visit in the rheumatology ward of Pisa University Hospital. Thus, these two criteria defined the inclusion criteria to identify users of bDMARDs tracked in the rheumatology ward. Indeed, this population included not only patients with rheumatoid arthritis but also those with other immune-mediated inflammatory diseases (extracted population). The information of diagnosis for these patients who had given their consent to participate to the study was extracted from the corresponding medical charts (reference). We tested the performance of the four index tests (algorithms) in identifying the true positive patients (i.e. patients with an assumed diagnosis of RA in the HAD who were actual RA patients in the reference)

bDMARD: biologic disease modifying anti-rheumatic drugs, HAD: Healthcare Administrative Database; ICD-9: international classification of diseases 9th revision; RA rheumatoid arthritis

SM Figure 2 Estimations of the first algorithm in the three analyses

Among patients with the first supply of bDMARD from 2014 to 2016 and at least one record of visit at the Rheumatology Unit of Pisa University Hospital from 2013 to the index date, the first algorithm, involving RA according to hospital discharge records or emergency department admissions (ICD-9 code, 714*) displayed low values (under 0.60) for sensitivity. This was observed in all the three analyses: the main analysis, the first sensitivity analysis, in which patients without diagnosis were excluded, and the second one evaluating subgroups of patients under and over 65 years old. In addition, high variability was found for the other estimations, like specificity, PPV and NPV, in the three analyses.

bDMARD: biologic disease modifying anti-rheumatic drugs, 95% CI: 95% confidence interval, ICD-9: international classification of diseases 9th revision, NPV: negative predictive value, PPV: positive predictive value; RA rheumatoid arthritis

SM Figure 3 Estimations of the second algorithm in the three analyses

Among patients with the first supply of bDMARD from 2014 to 2016 and at least one record of visit at the Rheumatology Unit of Pisa University Hospital from 2013 to the index date, the second algorithm, characterized by RA according to exemption code from co-payment (006), had good sensitivity, specificity, PPV and NPV over 0.70 in the main analysis. The first sensitivity analysis, not including patients with missing diagnosis, showed results consistent with those of the main analysis. The second sensitivity analysis displayed higher sensitivity and NPV in patients aged < 65 years and lower values for all the estimations in patients older 65 years than those obtained in the main analysis.

bDMARD: biologic disease modifying anti-rheumatic drugs, 95% CI: 95% confidence interval, NPV: negative predictive value, PPV: positive predictive value; RA rheumatoid arthritis

SM Figure 4 Estimations of the third algorithm in the three analyses

Out of patients with the first supply of bDMARD from 2014 to 2016 and at least one record of visit at the Rheumatology Unit of Pisa University Hospital from 2013 to the index date, the third algorithm included RA according to hospital discharge records or emergency department admissions (ICD-9 code, 714*) AND RA according to exemption code from co-payment (006). Very low sensitivity (0.37) was resulted in the main analysis. On the contrary, high specificity values (0.95) were observed. These findings were confirmed in the two sensitivity analyses, in which the values were consistent with those of the main analysis. Finally, high variability was observed for PPV and NPV when results of the three analyses were compared (the main, the first sensitivity excluding missing diagnosis, and the second one classifying patients based on their age in < or > 65 years) were compared.

bDMARD: biologic disease modifying anti-rheumatic drugs, 95% CI: 95% confidence interval, ICD-9: international classification of diseases 9th revision, NPV: negative predictive value, PPV: positive predictive value; RA rheumatoid arthritis
